# Effect of crisis resolution team treatment on crisis experience and crisis coping: a multicenter pre-post study in Norway

**DOI:** 10.1186/s12888-025-07491-y

**Published:** 2025-11-06

**Authors:** Katrine Høyer Holgersen, Nina Hasselberg, Johan Siqveland, Torleif Ruud

**Affiliations:** 1 Department of Psychology, Norwegian University of Science and Technology, Trondheim, Norway; 2 Nidelv Community Mental Health Center, Clinic of Mental Health, St. Olav’s Hospital, Trondheim, Norway; 3 Division of Mental Health Services, Akershus University Hospital, Lørenskog, Norway; 4 National Centre for Suicide Research and Prevention, Institute of Clinical Medicine, University of Oslo, Oslo, Norway; 5 Institute of Clinical Medicine, University of Oslo, Oslo, Norway

**Keywords:** Crisis resolution team (CRT), Crisis characteristics, Acute mental health, Crisis intervention, Community mental health services

## Abstract

**Background:**

Crisis resolution teams (CRTs) have been established in several high-income countries to improve access to mental health services and to prevent unnecessary inpatient admissions. General crisis theory is one of the foundations underpinning the development of CRTs. However, little research has been conducted on what characterises the situations leading to contact with these services and the crisis reactions during CRT care. This study aimed to describe characteristics and situations leading to contact with CRTs and to explore and predict possible changes in crisis experience and coping after CRT treatment.

**Methods:**

Participants of this Norwegian multicentre pre-post study included 546 CRT service users of 25 CRTs. No control group was included. The present study builds on data collected from service users and team workers. The main outcome variables were change in patient-reported crisis experience and crisis coping from start to end of treatment. We performed descriptive analysis of affected life domains, and linear mixed modelling to analyse how outcomes were associated with patients’ characteristics and treatment.

**Results:**

At treatment initiation, service users reported high scores of crisis experience and coping difficulties. Several life domains were affected, particularly emotional-life domains, such as mental illness, suicide risk, and loneliness. Lower levels of crisis experience, and enhanced coping abilities were observed within a timeframe of eight weeks or less. Although the current study design cannot rule out a specific causal relationship, recovery was nevertheless associated with service satisfaction, practical support, medication management, and quick access to help. Psychiatric symptoms at start, previous mental illness, and collaboration with wards were negatively related to a favourable outcome.

**Conclusions:**

CRT service users reported high levels of crisis experience and low levels of coping at treatment initiation. CRT treatment was associated with a decrease in severity and improvement in coping, although more severe mental health problems at the start were negatively related to recovery.

## Background

Crisis resolution teams (CRTs) were established to help people with acute mental illness to manage their situation without being admitted to psychiatric wards. CRTs can provide rapid assessment and offer intensive outpatient or home-based treatment if necessary. The model is most widespread in the United Kingdom (UK), but CRTs or similar teams have been established as part of acute mental health care in several other high-income countries [20]. A scoping review found research on such teams from USA, Denmark, Australia, the Netherlands, Germany, Ireland, South Korea, France, Switzerland, Spain, and Norway [19]. Similar health care teams are also found in Belgium [46], Malta [45], and Greece [27].

Many service users referred to CRTs experience a psychological crisis they cannot cope with, neither themselves nor with the help of their informal network, and the establishment of CRTs was based on (among other things) theoretical models developed to treat normally healthy individuals who are in a state of psychological crisis after experiencing a wide array of potentially traumatic events [37]. A precursor to such models was crisis intervention theory, formulated decades ago; where, early work by Gerard Caplan in the 1940 s and Eric Lindeman in the 1960 s contributed to knowledge about assessment and interventions after disasters [12]. Both in the traditional research field that describes human reactions following disasters and traumatic events, and in the literature on CRTs, crises are similarly described as ordinary human reactions to severe psychosocial stressors or situations where a person is overwhelmed and where their usual coping mechanisms break down [23, 29].

In more recent literature, qualitative studies on CRT users’ experiences have described typical crisis reactions, specifically in terms of feelings of chaos, loss of control, loss of energy, perceived helplessness, isolation, and difficulty coping with the duties of daily life [13, 26]. Other qualitative studies on the perspectives of clinicians have described loss of functioning, risk of injury, need for additional support to regain control, life events, social problems, and mental health problems among CRT service users [33, 49]. However, quantitative research on the experience and extent of the crises CRT patients face is scarce [14], consequently leading to a knowledge gap regarding the experience and development of crises and coping among CRT patients.

In the clinical field, the similarity between crisis management and the clinical interventions offered by CRT is more obvious. Providing elementary psychosocial support such as a supportive relationship which makes patients feel safe, accepted, and understood is crucial in CRT care [31]. A recent systematic review even identified six studies discussing facilitators and barriers when providing different psychological interventions within the frame of CRT treatment [2]. In research examining the structural organisation of CRT teams, availability and flexibility—i.e., gatekeeping teams which operate outside regular office hours, ability to provide frequent home visits or consultations, and seeking collaboration with the patients’ relatives/dependants—are emphasised as essential [15, 32, 51]. Recommendations for health care to be flexible, easily available, and individually adapted are also valid for interventions after disasters or other sudden adverse events [8, 48]. Similarly to what has been emphasized within the CRT field—that support from others is beneficial in cases of acute mental illness—research on disaster and traumatic stress shows that, although human responses to potentially stressful situations are influenced by many factors, the resources available within one’s social network, in particular have an important buffering effect [7, 35] Despite several similarities, differences may exist between extreme stressful or possible traumatic situations in general and the situations which lead people to seek help from CRTs. Crisis coping among CRT users may involve more complex needs, as previous or present mental health problems and life difficulties may add to the complexity of a crisis and limit one’s available coping resources [25, 37, 52]. Still, given the link between trauma and CRT’s theoretical and clinical underpinnings, the role of vulnerability and buffering factors from the research fields on crisis and trauma may also be relevant to explore among people seeking acute mental health care. To the best of our knowledge, this has not been previously investigated in quantitative studies.

### Aims

The present study aimed to explore situations which lead to contact with CRTs, as well as to predict possible changes in crisis experience and crisis coping from start to end of treatment. The research questions were: 1) What characterizes such crises, and what life domains are affected? 2) Are there changes in crisis experiences and crisis coping after CRT treatment? 3) What predicts the potential reduction in crisis experience and improved crisis coping after CRT treatment?

## Methods

### Design

This study is a part of a larger pre-post observational multicentre study of treatment outcome in Norwegian CRTs, with no control group [42]. Data were collected at the beginning and end of CRT treatment to examine potential associations between changes over time and the content of the intervention. Without a control group, the exact effect of the treatment cannot be determined. Several articles have been published [16, 17, 41, 43], but this is the first manuscript which describes the situations which led to contact with the team, crisis experience, and coping. The Network of Acute Mental Health Services in Norway (akuttnettverket.no) collaborated in the design of the study.

### Context

Secondary mental health services in Norway mainly consist of public services in four health regions. In 2016, South-Eastern Norway served 2.9 million (56%) inhabitants; Western Norway served 1.1 million (21%), Central Norway 0.7 million (14%), and Northern Norway served 0.5 million (9%) inhabitants [36]. The health regions provide general hospitals and other specialized health services, as well as mental health and substance abuse services from hospital inpatient units and community mental health centers (CMHCs) serving local catchment areas. The CMHCs have a variety of outpatient clinics, mobile teams, such as CRTs, and local inpatient units [40]. A report from 2015, showed that most of the 70 CMHCs had a CRT [44]. The CMHCs collaborate with primary health and social care run by the municipalities, such as general practitioners (GPs) and primary mental health and substance abuse care, as well as hospital units with a shared responsibility for the total mental health services. The municipalities have GPs on call 24/7 and primary care is given in the patient’s home when necessary, making primary care an important part of crisis and outreach services in Norway, even for patients with severe mental illness.

### Intervention

A recent publication from our project group gave a comprehensive account of the teams’ practices, detailing the intervention extensively [43]. We found that the Norwegian CRTs varied regarding accessibility and intervention components. Two thirds of the service users were having their first session on the day of referral or the following day. Intensity of treatment averaged 1.8 sessions during the first week, gradually decreasing thereafter. Six out of ten sessions took place in the teams’ locations while three of ten were held in the service users’ homes. Only two of ten sessions took place in the evening. Psychological interventions were provided to all service users, while family involvement, practical support and medication were less frequent. The average professions of the teams were 5.4 mental health nurses or nurses, 1.5 clinical psychologists, 1.3 psychiatrists or physicians specializing in psychiatry, and 1.0 social worker [43]. Two additional publications on the teams participating in our study provide more information about measured fidelity [16], and patients and their relatives experiences with care [17].

### Recruitment of CRTs

**Fig. 1:**
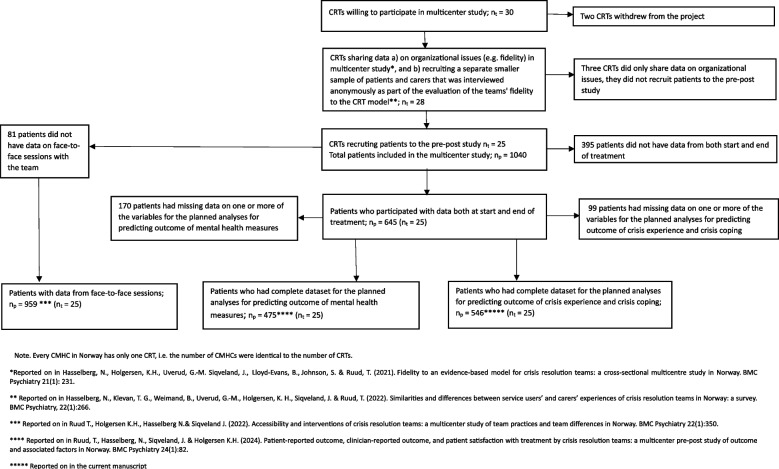
Flowchart of participating Crisis Resolution Teams (n_t_) and patients (n_p_) in the multicenter project on Norwegian CRTs

A thorough description of the recruitment and preparations of the participating CRTs were provided in a fourth publication from the study, which described changes in mental health from the start to the end of CRT treatment [41]. In short, the teams were recruited by an invitation sent in September 2014 to all the Norwegian health regions, and the CRTs showing interest participated in discussions on choosing the measures and practical procedures (i.e., by email, in a two-day network meeting of The Network of Acute Mental Health Services, and in a workshop during the final preparations). Based on interest shown by CRTs in the national network we aimed to recruit 30 CRTs. However, due to ongoing organizational changes, two teams withdrew their participation before the study started. Also, three of the remaining 28 CRTs did not recruit any service users to the study, due to various reasons. See Fig. 1 for a flowchart of participating CRTs.

Among the 25 teams recruiting service users to the study, 13 (52%) were located in the South-Eastern Norway health region, 6 (24%) in the Western Norway health region, 4 (16%) in Central Norway health region and 2 (8%) in the Northern Norway health region. Thus, reflecting the proportion of population served by the different geographical health regions as described above, and by representing CMHCs serving approximately half of the Norwegian population, the sample was considered representative. The data were collected between March 2015 and February 2016.

### Data collection

CRT members recruited participants during their first CRT consultation. Participants gave their written informed consent to participate, and service users and team members provided data from the start and the end of the treatment. Most of the 30 CRTs contributing to planning the study defined 4–8 weeks as the maximum duration of treatment. After establishing this consensus, and in line with the first RCT on CRTs measuring effect after eight weeks [23], the time frame for crisis treatment in the study was set to a maximum of eight weeks. Some teams treated service users for a longer period after the crisis treatment, and for any users who were not discharged within eight weeks, the questionnaire and closing form would be completed at the eight-week mark to obtain comparable data across the teams. In the previous publication describing the measured accessibility and interventions provided to 959 of the 1040 service users in the current project [43], we showed that duration for eight weeks or less applied to 92%. We did not separately analyze the 8% of participants with longer treatment period than eight weeks in the current study.

### Sample

The current study consisted of data from 546 participants (from 25 CRT teams) who reported complete data for all 29 independent variables at both the start and the end of treatment. This was 52.5% of the total sample of 1040 participants providing pre-data and 85% of the sample of 645 participants who shared data from both the start and the end of treatment. Sociodemographic descriptions of the participants are provided in Table 1.

**Table 1:** Socio-demographics and main diagnostic groups for participants (*n* = 546)

**Sex**	n	%
Women	331	60.6
Men	215	39.4
**Age**		
20 years	26	4.8
20–29	159	29.1
30–39	122	22.3
40–49	100	18.3
50–59	76	13.9
60–69	47	8.6
70–79	12	2.2
80–89	4	0.7
**Marital status**		
Married/Cohabitant	224	41.0
Single	229	41.9
Divorced	72	13.2
Widower/widow	12	2.2
Unknown	9	1.6
**Completed education**		
Not completed elementary school	8	1.5
Elementary school	113	20.7
High school	240	44.0
College/University	148	27.1
Unknown	37	6.8
**Main source of income**		
Employed	215	39.4
Student’s funding	29	5.3
Unemployment benefits	11	2.0
Sick leave/rehabilitation	111	20.3
Disability leave	95	17.4
Retired	29	5.3
Other social security	4	0.7
Social welfare	11	2.0
Supported by others	20	3.7
No income	9	1.6
Unknown	12	2.1
**Main diagnosis at end of treatment**		
Psychosis or bipolar disorder	46	8.4
Depression	173	31.7
Anxiety	178	32.6
Substance use disorders	20	3.7
Personality disorders	27	4.9
Other disorders	19	3.5
No diagnosis	83	15.2

### Measures

#### Outcome measures

The two primary outcomes were change in patient-reported experience of crisis severity and crisis coping from start of treatment to follow-up at 8 weeks (pre-value minus post-value, with positive change indicating improvement). These were measured using the Crisis State Assessment Scale (CSAS; [29]). The scale has not previously been used in a patient population within CRT and was translated into Norwegian by the principal investigator of the multicenter study (TR). In the original version of the CSAS, respondents were introduced to the questionnaire as follows: "This questionnaire is designed to measure the way you feel as a result of a traumatic event or situation that has happened within the last month," and were then asked to briefly describe the event or situation that had triggered the current reaction. When designing the current study, it was considered most appropriate to introduce the scale with the following question, given that the inclusion criterion for referral to a Crisis Resolution Team was being in a crisis situation: "How do you experience the situation you are in?".

The response scale was adapted from “how often” to “to what extent” to better fit the experiences of a short-time ongoing crisis and shortened from the original seven-point scale to a five-point scale to align with the other questions in the questionnaire. Items were scored from one to five, with higher scores indicating higher crisis severity or poorer coping. In the original publication, [29] the CSAS scale showed good psychometric properties for the original two subscales; experience of crisis, item 1–5 (Cronbach’s alpha 0.85), and crisis coping, item 6–10 (Cronbach’s alpha 0.84). In the present study, principal component factor analysis with varimax rotation and Kaiser’s criterion of eigenvalue 1 or more identified the same two factors of CSAS, except that item 5 shifted from the first to the second factor: experience of crisis, under items 1–4 (Cronbach’s alpha 0.88), and crisis coping, under items 5–10 (Cronbach’s alpha 0.82).CSAS is considered to cover aspects of CRT users' experiences that go beyond what was related to psychiatric symptoms. To the best of our knowledge, we are the first to apply a self-report measure designed to assess crisis experiences after stress in a population with acute mental illness.

#### Independent variables

*Life events or circumstances* associated with the initial contact with the CRT were assessed at treatment initiation where the participants were asked: “Does the situation and your contact with the acute team relate to the following events or circumstances?”. Using a five-point Likert scale, they responded whether this applied from “not at all” (1) to “a very large extent” (5) for problems related to: housing/economics, work/education, physical illness, mental illness, relationship/marital problems, loneliness, accidents or deaths, threats/violence/ abuse, self-harm/suicidality, or substance use.

*Perceived social support* from family and friends (i.e., informal network) was measured using the Crisis Support Scale (CSS; [24]) at the start and the end of treatment. The scale comprised seven items, each scored on a seven-point Likert scale from “never” (1) to “always” (7), with higher scores indicating more support. Cronbach’s alpha in the present sample was 0.68.

*Symptoms of psychological distress* at the start and the end of treatment were measured using the Clinical Outcome in Routine Evaluation Outcome Measure (CORE-10; [4]). This is a short form of the CORE-OM (CORE-OM; [11]) and is a self-administered questionnaire with items related to mental problems, well-being, functioning, and risk during the preceding week. All items are scored from “never” (0) to “almost all the time” (4), with higher scores indicating more serious problems. Both CORE-OM and CORE-10 have shown good psychometric properties [4, 10, 11]; in the present sample, Cronbach’s alpha was 0.75 at treatment start and 0.88 at treatment end.

*Patient satisfaction* was assessed after CRT treatment using the Client Satisfaction Questionnaire (CSQ-8; [28])*.* The eight-item self-report questionnaire captures quality of the service and general satisfaction, and it is scored on a four-point scale from 1 to 4, with higher scores indicating higher satisfaction. Cronbach’s alpha in the present sample was 0.90.

Finally, two single items were recorded in the patients’ self-report at treatment start: *a) Duration of crisis or difficult situation*, rated on a four-point scale, indicated how long time the situation had been going on, from “last 24 h” (0) to “several weeks” (3); *b) Type of event* was rated on a four-point scale, from “sudden event” (0) to “long lasting situation” (3).

Demographic variables—such as age group, sex, and housing situation—as well as current diagnosis, previous mental health problems, and contact with the CRT was reported by therapists at the start of treatment. To predict the possible role of accessibility and interventions provided by CRTs during treatment, we drew on the findings on teams’ level of accessibility and fidelity to the CRT model taken from two recently published papers from the same project [16, 43]. In the present study, the accessibility variables included the possibility to self-refer, response time (from received referral to first session), proportion of participants with their first session on referral day, number of sessions per service user, proportion of sessions outside CRT location, proportion of sessions outside office hours, duration of sessions, duration of crisis interventions (in weeks), and intensity of crisis intervention (sessions per week). Intervention components included practical support, psychological interventions, family involvement, medication management, collaboration with inpatient services (mostly CRTs preparing and implementing inpatient admissions), and collaboration with general practitioners (GPs) and municipal primary care services.

### Data analyses

To describe crises and affected life domains, we used descriptive analyses to calculate frequency, mean (M), and standard deviation (SD). To explore changes in participants’ experience of the crisis and crisis coping, we used paired *t*-test and Cohen’s d to describe effect sizes for changes in each item.

The associations between the independent variables and each of the two outcome variables (crisis experience and crisis coping) were analyzed using linear mixed modelling with estimation of the teams’ level proportion of the total variance of the dependent variable (intraclass correlation coefficient, or ICC). Three models were defined for each outcome variable: Model A, for association with variables in the situation at the start; Model B, for association with the different treatment elements provided; and Model AB, for association with both the situation at the start and the treatment provided.

In Model A, we included 12 independent variables for the situation at the start: three were sociodemographic (age, sex, and living alone), four were health variables (diagnosis of psychosis, previous mental illness, previous team contact, and self-reported mental health problem; CORE-10) and five variables described the current crisis leading to CRT contact (suddenness and duration of the situation, crisis experience (CSAS items 1–4), crisis coping (CSAS items 5–10), and crisis support (CSS)).

In Model B, we included 17 independent treatment variables: nine accessibility variables (self-referral, response time, first session occurring on referral day, number of sessions, sessions outside CRT location, sessions outside office hours, duration of sessions, treatment duration, and number of sessions per week), six variables on intervention components (practical support, psychological interventions, family involvement, medication management, collaboration with inpatient services, and collaboration with GP and primary care), and one variable on patient satisfaction at treatment end (CSQ-8). The one variable on teams’ level was the mean total fidelity, provided in a previous publication in the same project [16].

Model AB included all 29 independent variables in Models A and B and thus consisted of both the variables on the situation at treatment start and the variables on the services provided by the CRTs.

We used linear mixed models to analyze the associations of factors with each of the two outcomes. All the independent variables were included as fixed effects, although total fidelity was included as a random effect with unstructured covariance. The models were reduced by eliminating variables one by one based on lowest z-value and highest *p*-value and comparing the models using the Bayesian Information Criterion (BIC), where a lower value indicates a better model. We present the results for each model with the lowest BIC value.

We used STATA version 17 to conduct the linear mixed model analyses and SPSS version 27 for the remaining statistical analyses.

## Results

### Current situation leading to contact with CRTs

**Fig. 2:**
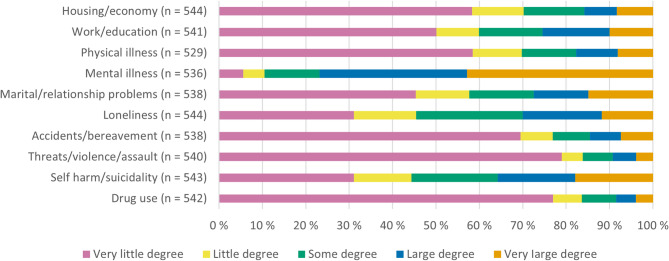
Problems in life domains or circumstances at start of CRT treatment (*N* = 546)

Figure 2 shows the patient-reported impact of the different life domains on the current situation which led to their contact with the CRT. On average, the respondents reported two to three life domains being largely or very largely associated with the crisis (M = 2.57, SD = 1.61). Emotional problems were most often reported; problems related to mental health were reported to a high or very high degree for *n* = 412 (76.9%), followed by issues related to self-harm/suicidality (*n* = 194, 35.8%), loneliness (*n* = 163, 30.0%), marital/relationship problems (*n* = 148, 27.5%), work/education (*n* = 138, 25.5%), physical illness (*n* = 94, 17.7%), housing/finances (*n* = 86, 15.8%), accidents or deaths (*n* = 78, 14.5%), threats/violence/assault (*n* = 50, 9.3%), and substance use (*n* = 46, 8.5%).

Table 2 shows participants’ perceptions of the current situation and their perceived social support reported at the start of treatment

**Table 2:** Duration of crisis, type of event and perceived social support by Crisis Support Scale (CSS) at start of CRT treatment (*n* = 546)

**Duration of crisis**	n	%
The last 24 h	10	1.6
The last days	52	9.5
The last 1–2 weeks	104	19.0
Several weeks	380	69.6
**Type of event**		
Sudden	69	12.6
Rapid deterioration	74	13.6
Gradual aggravation	168	30.8
Lasted for a long time	235	43.0
**Perceived social support at admission**	**Mean**	**SD**
CSS 1: Does anyone listen when you need to talk about the situation? (*n* = 545)	4.96	1.65
CSS 2: Have you had contact with others with similar experiences? (*n* = 543)	2.79	1.63
CSS 3: Can you talk about thoughts and feelings? (*n* = 544)	4.54	1.62
CSS 4: Does anyone show compassion and support? (*n* = 546)	5.08	1.51
CSS 5: Does anyone give you practical help? (*n* = 538)	4.05	1.80
CSS 6: Have you felt let down by someone you expected to support you? (*n* = 543)	3.55	1.83
CSS 7: All in all, are you happy with the support you receive from family/friends? (*n* = 546)	4.25	0.97
**Perceived social support admission (CSS- total scale)**^a^	4.25	0.97

^a^Scored on a seven-point scale from 0: never to 7: always; CSS: Crisis Support Scale

For most participants, the crisis had lasted for a while: almost nine out of ten (*n* = 484; 88.6%) reported that it had lasted for one or more weeks. One out of four participants (*n* = 143; 26.2%) described the situation leading to their contact with the CRT as occurring suddenly or as rapidly deteriorating.

Overall, participants reported high satisfaction with perceived support from family and friends. Over half (*n* = 302; 55.3%) reported they often (*n* = 120; 22.0%), very often (*n* = 98; 17.9%), or always (*n* = 84; 15.4%) were satisfied with received support. On the other single items, more than a third reported to receive support often, very often, or always, and the mean score on each item was high. The exception being that only about one in seven (*n* = 78; 14.3%) reported having had contact with others in the same situation. Among these, 9.2% (*n* = 50) reported having had such contact often, 3.1% (*n* = 17) very often, and 2.0% (*n* = 11) always.

### Changes in crisis experience and coping with crisis after CRT treatment

Table 3 shows that high scores on crisis experience were reported at treatment start. The total score fell from a mean of 3.99 (*SD* 0.69) at treatment start to a mean of 3.03 (*SD* 0.92) at treatment end. The change on the total scale as well as on the subscales of crisis experiences and crisis coping were all highly significant. In terms of clinical significance, the number of service users reporting mean scores of 3 or less rose from 11.5% (n = 63) at start of treatment to 49.6% (n = 271) at end of treatment. Large effect sizes (Cohen’s d ≥ 0.90) were detected for the total scale and the two subscales. For most single items, Cohen’s d ranged from 0.73 to 0.98—except for item 9 and 10, which demonstrated a medium/medium-to-low effect size (*d* = 0.51 and *d* = −0.39).

**Table 3:** Change in crisis experiences and crisis coping after CRT treatment (*N* = 546)

	n	After first meeting		After last meeting		Mean change				Effect size	
		*M*	*SD*	*M*	*SD*	*M*	95% CI	*t*	*df*	Cohen’s d	95% CI
**CSAS 1–10 total scale**^a^	546	39.83	6.89	30.25	9.19	0.96	8.86–10.30	26.18***	545	1.12	1.01−1.23
**CSAS 1–4 crisis experiences**^b^	546	4.38	0.71	3.30	1.01	1.09	1.00–1.17	22.83***	545	1.10	1.00−1.21
**CSAS 5–10 crisis coping**^b^	546	3.72	0.77	2.84	0.95	0.87	0.80−0.95	26.18***	545	0.98	0.88−1.08
1. I think about the situation when I don’t want to	545	4.33	0.85	3.25	1.05	1.08	0.98−1.17	22.76***	544	0.98	0.87–1.08
2. I feel like the situation throws my life off balance	542	4.25	0.95	3.14	1.15	1,10	1.00–1.20	21.42***	541	0.92	0.82–1.02
3. I feel like my physical or emotional well-being is threatened by the situation	540	4.49	0.75	3.48	1.09	1.01	0.92–1.11	21.36***	539	0.92	0.82–1.02
4. The situation is very distressing to me	542	4.48	0.77	3.32	1.22	1.15	1.05–1.25	21.97***	541	0.94	0.84−1.04
5. The situation makes me feel like I am going crazy	541	3.17	1.38	2.21	1.22	0.96	0.86–1.07	17.46***	540	0.75	0.66−0.85
6. I feel like I don’t have the resources and/or energy to deal with the situation	545	4.06	0.96	3.14	1.18	0.92	0.82–1.03	17.01***	544	0.73	0.63−0.82
7. I don’t know what to do to make this situation manageable	541	4.05	1.00	2.91	1.22	1.14	1.03–1.25	20.13***	540	0.87	0.77−0.96
8. I feel like I cannot handle the situation	545	3.98	1.00	2.83	1.19	1.15	1.04–1.26	21.17***	544	0.91	0.81–1.01
9. I don’t deal well with situations like this one	544	3.76	1.03	3.17	1.04	0.59	0.49−0.68	11.92***	543	0.51	0.42−0.60
10. I am confident that I can cope with the situation	545	2.73	1.10	3.19	1.10	−0.46	−0.56 – (−0.37)	−9.30***	544	−0.40	−0.49- (−0.31)

*CSAS* Crisis State Assessment Scale; ^a^mean sum for total scale, ^b^mean item for subscales, the scores for item 10 are reversed; *** *p* <.001

### Factors associated with reduction in crisis experience and improved coping after CRT treatment

Table 4 presents the variables significantly associated with patient-reported reduction in crisis experience in the linear mixed models. Eight of the 29 independent variables were included in Model AB with the lowest BIC value. Patient-reported symptom score at treatment start (CORE-10) and collaboration with mental health wards were negatively related to reduction in crisis experience. By contrast, crisis experience reported at treatment start, first session occurring on day of referral, treatment duration, practical support, medication management, and patient satisfaction were positively associated with reduction in crisis experience. Treatment intensity and family involvement were positively associated with the outcome in Model B. Absolute values for duration of treatment were: 1–2 weeks (*n* = 288; 52.7%), 3–4 weeks (*n* = 111; 20.3%), 5–6 weeks (*n* = 69; 12.6%), 7–8 weeks (*n* = 49; 9%) and more than 8 weeks (*n* = 29; 5.3%). For treatment intensity (meetings pr week during treatment) the data showed that in average, participants pr week had 1.0–1.9 meetings (n = 381; 69.8%), 2.0–2.9 meetings, (*n* = 126; 23.1%), 3.0–3.9 meetings (*n* = 29; 5.3%) or 4 or more meetings pr week (*n* = 10; 1.8%).

**Table 4:** Association of background and treatment variables with reduction in crisis experience at end of treatment (*N* = 546). Linear mixed effects models

**Variables**	**Coefficient**	**CI 95% (lower, higher)**		***p***
**Model A: Situation at start of treatment**				
Psychiatric symptoms start of treatment (CORE-10)	−0.27	−0.42	−0.12	< 0.001
Crisis experience start of treatment (CSAS 1—4)	0.59	0.46	0.72	< 0.001
- Constant	−0.81	−1.29	−0.32	0.001
Team level variance: 9.2%				

**Variables**	**Coefficient**	**CI 95% (lower, higher)**		***p***
**Model B: Treatment provided**				
Treatment duration (weeks)	0.04	0.01	0.07	0.007
Treatment intensity (meetings per week)	0.09	−0.03	0.20	0.128
Practical support	0.56	0.16	0.97	0.006
Family involvement	0.15	−0.18	0.48	0.364
Medication management	0.45	0.10	0.80	0.012
Collaboration with mental health wards	−0.30	−0.77	0.16	0.198
Client satisfaction end of treatment (CSQ-8)	0.58	0.41	0.75	< 0.001
- Constant	−1.31	−1.94	−0.68	< 0.001
Team level variance: 7.0%				

**Variables**	**Coefficient**	**CI 95% (lower, higher)**		***p***
**Model AB: Situation at start of and during treatment**				
Psychiatric symptoms start of treatment (CORE-10)	−0.24	−0.38	−0.10	0.001
Crisis experience start of treatment (CSAS 1–4)	0.56	0.44	0.69	< 0.001
First session on day of referral	0.19	0.04	0.35	0.011
Treatment duration (weeks)	0.03	0.01	0.06	0.018
Practical support	0.63	0.26	1.01	0.001
Medication management	0.39	0.06	0.71	0.019
Collaboration with mental health wards	−0.44	−0.88	−0.01	0.044
Client satisfaction at end of treatment (CSQ-8)	0.50	0.34	0.66	< 0.001
- Constant	−2.80	−3.49	−2.10	< 0.001
Team level variance: 8.3%				

Table 5 presents the variables significantly associated with patient-reported improved crisis coping at the end of treatment, which include six of the 29 independent variables from the model AB with the lowest BIC value. Previously known mental illness, patient-reported symptom score at treatment start (CORE-10), and collaboration with mental health wards were negatively associated with improved coping. Poorer crisis coping at treatment initiation, practical support, and patient satisfaction were positively associated with improved crisis coping at treatment end. No additional factors were kept in either Model A or Model B.

**Table 5:** Association of background and treatment variables with improved crisis coping at end of treatment (*N* = 546). Linear mixed effects models

**Variables**	**Coefficient**	**CI 95% (lower, higher)**		***p***
**Model A: Situation at admission**				
Previously known mental illness	−0.19	−0.33	−0.05	0.007
Psychiatric symptoms start of treatment (CORE-10)	−0.31	−0.46	−0.17	< 0.001
Crisis coping at start of treatment (CSAS 5–10)	0.60	0.48	0.71	< 0.001
- Constant	−0.43	−0.77	−0.08	0.015
Team level variance: 7.3%				

**Variables**	**Coefficient**	**CI 95% (lower, higher)**		***p***
**Model B: Treatment provided**				
Practical support	0.41	0.05	0.78	0.028
Collaboration with mental health wards	−0.44	−0.85	−0.03	0.035
Client satisfaction end of treatment (CSQ-8)	0.60	0.45	0.75	< 0.001
Constant	−1.22	−1.76	−0.67	< 0.001
Team level variance: 4.2%				
**Model AB: Situation at start of and during treatment**				

**Variables**	**Coefficient**	**CI 95% (lower, higher)**		***p***
Previously known mental illness	−0.17	−0.30	−0.04	0.009
Psychiatric symptoms start of treatment (CORE-10)	−0.30	−0.44	−0.17	< 0.001
Crisis coping at start of treatment (CSAS 5—10)	0.61	0.50	0.72	< 0.001
Practical support	0.40	0.07	0.73	0.016
Collaboration with mental health wards	−0.69	−1.06	−0.32	< 0.001
Client satisfaction end of treatment (CSQ-8)	0.60	0.46	0.73	< 0.001
Constant	−2.63	−3.21	−2.04	< 0.001
Team level variance: 8.4%				

In the three models (A, B, and AB), the teams’ level variance in predicting reduced crisis experience ranged from 7.0% to 9.2%. For the models predicting improved crisis coping, the range was from 4.2% to 8.4%.

To address concerns around possible multicollinearity and model stability and investigate the possible bias introduced by having many independent variables that might introduce collinearity, we performed separate multiple regression analysis with additional collinearity statistics. We found all Variance Influence Factors (VIF) values to be well below the suggested threshold of 5.

## Discussion

The main findings of the present study revealed that service users seeking help from CRTs reported high scores on crisis experience and difficulties in coping with crisis at treatment initiation, as well as a large reduction in crisis experience and improved crisis coping over eight weeks or less. The crisis had often developed over some time, and several life domains were affected—especially emotional issues, in the form of mental health problems, suicide risk, and loneliness. Current and previous mental health problems had a negative association with decreased crisis experience and improvement in coping.

### Affected life domains and characteristics of the crisis leading to contact with CRTs

The findings showed that several contextual factors contributed to the patients’ current situation. For most CRT service users, mental illness, suicide risk, loneliness, and marital problems were more often rated as problematic, but there were also problems with practical issues such as housing, economy, work and education. The role of contextual factors for patients seeking mental health care is in line with a study by Probst et al. [38], which suggested that therapists be attentive to changes in clients’ social support and experience of life events occurring during therapy, as this is particularly important to avoiding negative outcomes. The current results emphasize the importance of designing acute mental health care services broadly enough to help users find solutions to both specific challenges such as housing, economics or work and education, but also addressing more emotional issues. Previous trauma-related research has documented that subjective appraisal of an event—such as perceived life threat, peri-trauma fear, comorbid psychological problems, social withdrawal, and thought suppression—subsequently impact adaption and increase the risk for posttraumatic stress [50]. Sharing hope and relief to reduce or moderate patients’ subjective suffering during a mental health crisis is likely an important clinical tool. Psychiatric treatment for suicidal inpatients needs to prioritise physical safety through observation and containment [6]; nevertheless, the current findings suggests that it could be appropriate to increase the emphasis on patients’ desires to receive psychological interventions, such as making sense of and addressing difficult emotional issues in terms of suicidality, even in the midst of a life crisis. This is in line with the findings from Awenat et al. [3], with a similar suggestion drawn from a qualitative study on suicidal inpatients’ experiences and expectations. Although there is currently limited knowledge, it seems possible to implement psychological interventions within the framework of an acute setting in a CRT [2].

The high scores on emotional aspects of crisis suggest that helping CRT service users reconnect with others may be important. A recent literature review showed that, although the importance of the professional relationship between professionals and service users in crisis is well-researched and documented in several other contexts, there is limited empirical evidence regarding its significance within research on CRT treatment [47]. Further, a study from the UK showed that persistent severe loneliness was associated with worse personal recovery among CRT service users [30], while a self-management intervention facilitated by peer-support workers after acute mental illness could reduce readmissions [22]. Moreover, one may encounter normalising and validating reactions when meeting others in similar situations, which is likely to contribute to reducing feelings of shame and increasing feelings of acceptance and shared hope. For several decades, access to social support has been recognised as one of the most salient factors in a crisis situation, and the absence of such support appears to predict development of mental illness following various situations that may trigger typical crisis reactions, such as after exposure to possible traumatic events [7]. While our participants overall reported high satisfaction with the support from family and friends (see the CSS results), very few had shared their experiences with others in a similar situation. In a CRT context, both therapeutic interventions applied within the therapeutic relationship along with strengthening or reactivating the service user’s social network may decrease isolation and despair by increasing feelings of connectedness with others.

### Reduced crisis experience and improved crisis coping after CRT treatment

The participants seemed to understand and assess their current challenges within a framework which measured crisis experience and lack of coping with crisis. Initial scores were high on both outcomes, and associations with large effect sizes were observed following the treatment period. The development seems parallel to the positive change in mental health status reported in our previous publication [41]. Pearson's correlation between CORE-10 and CSAS crisis experience at baseline was 0.568. Although this relatively strong correlation shows partial overlap, it does not exceed 0.70 which would cause them to be mutually exclusive in analyses. Our findings suggest that concepts derived from crisis theory may improve clinicians’ understanding of many CRT service users. For one thing, having the first session on the day of referral and patient satisfaction were related to positive outcomes. Accessibility and flexibility is in line with recommendations found in the literature on interventions after disasters or other sudden adverse events [8, 48]. The participants’ description of the crisis in the CSAS is also in line with those from previous qualitative studies exploring CRT service users’ and team workers’ views and reflections on the crisis leading to contact with the CRT [13, 26, 33, 49]. Given the increased focus on psychological interventions after crises in general, it is surprising that studies on CRTs have not included measures of the crisis leading to contact with the teams.

Most participants in the current sample were diagnosed with anxiety or depression, and to investigate representativeness of service users in the CRTs, 11 teams reported some characteristics of the included service users and the overall caseload of the teams for three months after the data collection [42]. On average one third of the CRTs service users were included in the multicentre study, and with a smaller percentage of those who had psychosis and/or were admitted to inpatient units. It is unclear how many excluded patients were not invited to participate or declined to participate, but it is likely that service users with more severe mental problems were less likely to be included. Most service users were able to complete the questionnaire at treatment start, as such completed questionnaires were only missing for 3.4% of the 1,018 services users with completed assessments by the team at the treatment start. Testing how representative the current sample included in this paper was compared to the total sample in the multicentre study, we found small differences at the treatment start between these 546 service users and the remaining 472 where the assessments were not complete. There were no significant differences regarding age, sex, crisis experience (CSAS 1–4) or self-reported psychiatric symptoms (CORE-10). The current sample had significantly slightly less coping with crisis (CSAS 5–10) and significantly higher clinician-reported psychiatric problems (HoNOS) than those not included in the current sample.

As CSAS demonstrated satisfactory internal reliability and sensitivity to change with the present sample, the questionnaire may be appropriate for use in future research. We emphasize, however, that research on other samples from CRTs or adjacent acute mental health services is needed.

### Factors associated with reduction in crisis experience and improved crisis coping after CRT treatment

Although measured only at the end of treatment, patient satisfaction was strongly positively associated with reduction in crisis experience as well as with improved coping. Furthermore, practical support, medication management, and accessibility (first session occurring on day of referral) were positively related to reduced crisis experience and to improved crisis coping. The regression coefficient for the positive association between treatment duration and reduction in crisis experience was low, indicating that other aspects of the treatment rather than duration may be more important. An alternative interpretation may be that patients with more severe mental disorders need a longer course of treatment than 4–8 weeks in order to see an effect. In a further focus on patients with the most serious mental health problems, both patient-reported psychiatric symptoms at baseline (CORE-10) and collaboration with psychiatric wards (i.e., CRTs preparing and implementing inpatient stays) had the most pronounced negative associations with reduction in crisis experiences and improved coping. In addition, previous mental illness was negatively associated with improved crisis coping. An understanding of the findings which provide nuance to the image above may be that for those with more severe problems, the crisis experience may not be as relevant because the patients have such serious ailments.

Although many service users benefitted from CRT treatment, as outlined above, our results show that service users with more severe problems did not show the same reduction in crisis experience and improvement in coping. Because medical and practical support was positively related to outcomes, the findings imply that CRTs should be able to provide these measures to users in need of such assistance. Need for admission to inpatient wards may be due to more severe illness or, alternatively, the fact that the Norwegian CRTs in this sample had low fidelity scores on (among other things) opening hours, intensity of contact, providing medication, and providing practical support [16, 41].

Psychological interventions were the most common intervention, probably because many clinical psychologists work in the Norwegian CRT teams. However, the psychological interventions were not kept in the final models for the outcomes. This is difficult to interpret but the classification of psychological interventions was rather broad, consisting of “Working through thought/feelings”, “Clarifying/sorting the situation”, “Providing information”, and “Psychotherapy” [43]. This means that the interventions were not specified by subtype of psychological orientations. As a result, the broad category might have masked a possible effect.

A recent scoping review found very limited literature on the use of evidence-based psychological approaches in the CRT context [19], but a specific trauma-focused approach, EMDR [39], and a cognitive-based intervention [9] showed promising results. Other therapeutic interventions used in CRTs include a skill-based intervention [34] and studies exploring factors such as the role of relational and common factors in a CRT context [5]. Although the short time frame of most CRT treatments limits the extent to which this service should initiate complex psychological interventions [21], recent guidelines from the Association of Clinical Psychologists UK underscore that psychological interventions have thus far not been accessible in acute adult mental health care [1]. Further development and testing of psychological interventions adapted to CRT treatment may be important for the future. Alternatively, the observed reduction in crisis experience and the observed increase in coping—with mostly high effect sizes—at the end of treatment may indicate that elements of general psychological crisis intervention already seem to be a part of CRT treatment. Although not studied in the present sample, an aim to promote a sense of safety, calm, self-efficacy, connectedness, and hope has been regarded as an essential element for people in general who are experiencing intense states of crisis [18]. Given the current results demonstrating the relevance of general crisis theory in CRT care, further studies could explore how these principles might also apply to service users with more severe issues or those admitted to inpatient mental health care. This could, for example, build on the work from a recent overview study that, through six publications, provides knowledge about the factors that promote and hinder the implementation of psychological interventions within CRTs [2].

### Strengths and limitations

The main strength of the present study is that it was the first in the field to include self-reported measures from several hundred CRT service users on aspects of the situations or events leading to their contact with acute mental health care, and their change in crisis experience and crisis coping after treatment. However, due to the observational study design, a possible limitation of the study is that we cannot rule out alternative explanations for the observed changes, such as regression to the mean, spontaneous recovery, or the unlikely but possible influence of concurrent treatments. A further related limitation of the study design is the possibility of residual confounding, as treatment components such as practical support and medication were not randomly assigned but provided based on clinical indication. Consequently, the estimated regression coefficients may not reflect causal relationships, and the reported associations are conditional on the measured covariates. It is plausible that the direction of this confounding could lead to either an overestimation or underestimation of treatment effects. In the absence of random assignment, causal inference remains limited, and these considerations should be considered when interpreting the findings. A further limitation of the study relates to the translation of the CSAS. Several adaptations were made from the original version to ensure relevance for participants seeking help from a CRT, rather than responding to a single specific incident. These modifications may have affected the validity of the scale. However, a strength of the study is that it included a measure designed to assess crisis experiences following stress in a population with acute mental illness

Although the data was collected 9–10 years ago, and a limitation is that the data therefore might be considered a bit old, it is a strength that no quantitative research has previously been published on such data. One major limitation is the unknown variation across CRTs with respect to the proportion of services users who were included, and that only 11 of 25 (44%) teams were able to report some characteristics of the included service users and the overall caseload of the teams when contacted after the data collection to investigate this. Also, the follow up report from the 11 teams indicates that it is likely that some patients with the highest symptom burden were not included in the study, which adds to the limitations. However, the fact that only 3.4% of the participants included were unable to complete self-reports at the start adds to the strengths. As almost half of the CRTs in urban and rural areas throughout Norway participated; thus, the study was nationally representative, which also adds to its strengths. Another limitation may be that the fidelity to the CRT model varied across the Norwegian teams in the present study [16]. Because several CRTs are not set up to be able to offer sufficient intensive follow-up in the event of a serious mental illness, the sample in the current study may not reflect the situation for the most distressed patients. More research is needed to explore variables related to crisis experiences and crisis coping in further studies on CRTs or similar interventions for people with acute mental illness.

### Conclusions and clinical implications

Although CRT service users initially reported high scores on crisis experiences and coping difficulties, their strain eased significantly after receiving CRT treatment. However, such improvement was less pronounced for those with more serious illness. In the total sample, emotional-life domains, such as mental illness, suicide risk, and loneliness, seemed the most crucial aspects of the crisis. These findings underscore relational factors as important in acute mental health care and concept of crisis as valuable when approaching people seeking help from CRTs. However, practical support and medication appeared crucial for improvement for the services users with more severe mental symptoms and illnesses.

## Data Availability

The dataset used and analyzed during the current study may be made available from the corresponding author on reasonable request.

## Abbreviations:

BIC: Bayesian information criterion
CMHC: Community mental health centers
CORE-10: Clinical outcome in routine evaluation 10
CRT: Crisis resolution team
CSS: Crisis Support Scale
CSAS: Crisis State Assessment Scale
CSQ-8: Client Satisfaction Questionnaire
EMDR: Eye Movement Desensitization and Reprocessing
GP: General Practitioner
M: Mean
SD: Standard deviation
